# Proteomic and metabolomic analyses reveal the novel targets of spermine for alleviating diabetic cardiomyopathy in type II diabetic mice

**DOI:** 10.3389/fcvm.2022.1022861

**Published:** 2022-10-14

**Authors:** Jian Sun, Jiyu Xu, Yong Liu, Xiaoyi Xu, Shumin Zhang, Yankun Hao, Yitong Lin, Yue Han, Feiya Li, Hui Yuan

**Affiliations:** ^1^School of Basic Medical Sciences, Mudanjiang Medical University, Mudanjiang, China; ^2^School of Medical Imaging, Mudanjiang Medical University, Mudanjiang, China; ^3^Research Department, Animal Research Institute, Mudanjiang Medical University, Mudanjiang, China; ^4^The First Clinical School of Medicine, Mudanjiang Medical University, Mudanjiang, China; ^5^School of Stomatology, Mudanjiang Medical University, Mudanjiang, China; ^6^Department of Laboratory Medicine and Pathobiology, Sunnybrook Research Institute, University of Toronto, Toronto, ON, Canada

**Keywords:** diabetic cardiomyopathy, spermine, proteomic analysis, bioinformatic, PRKG1

## Abstract

Diabetic cardiomyopathy (DCM) is one of the most serious complications of diabetes. Recent cardiology studies suggest that spermine has a cardioprotective effect. Here, we used proteomic and metabolomic analyses to reveal the underlying research targets in a type II diabetic (T2D) mouse model treated with spermine. Left ventricular tissues from nine mice (Control group, three; T2D group, three; T2D+SP group, three) were excised and analyzed. Quantitative analysis of the global proteome and metabolome was performed using the 4D label-free technique and untargeted metabolomics, respectively, and differentially expressed proteins (DEPs) and metabolites were used to perform bioinformatic analyses. A total of 169 DEPs were identified in T2D/Control group, including 115 upregulated and 54 downregulated proteins. Furthermore, 16 DEPs were identified in T2D+SP/T2D group, where these DEPs were found highly enriched in the cellular, metabolic processes, biological regulation, response to stimulus, and immune system process. The results of association analysis between proteomics and metabolomics showed that SP could affect the production of 51 metabolites by regulating the expression of 16 DEPs in the T2D+SP/T2D group. We also found that PRKG1 was closely related to the expressions of 10 overlapping metabolites between db/db and SP-treated mice. Our findings provide insights into the underlying mechanisms for DCM and suggest the potential applicability of utilizing spermine on protecting against DCM-associated cardiac function deterioration.

## Introduction

Diabetic cardiomyopathy (DCM) is a disorder in which the structure and the function of the myocardium are directly impaired following diabetes and is different from myocardial lesions caused by other heart diseases including coronary disease, valvular disease, or risk factors including hypertension (1). DCM is characterized by an obvious diastolic dysfunction at the early stage and a serious systolic dysfunction at a later stage, these eventually develop into arrhythmia, heart failure, shock, and even sudden cardiac death (2). DCM myocardial tissues display serious metabolic abnormalities and obvious microvascular lesions, which further develop into diffuse necrosis with potential subclinical abnormal cardiac function including ventricular stiffness, myocardial hypertrophy, and myocardial fibrosis (3). However, the exact mechanism underlying DCM remains unknown. Therefore, uncovering accurate and effective therapeutic targets is urged.

Polyamines (PAs) are small, branched-chain cationic molecules derived from linear amino acids and are widely distributed in all types of mammalian cells. Naturally occurring polyamines include spermine (SP), spermidine (SPD), and putrescine (PU) (4). SP participates in various biological processes of the organism, including regulation of DNA synthesis, cell cycle, cell proliferation and differentiation, aging, endoplasmic reticulum stress, oxidative stress, and ion channel switching, and also has anti-apoptosis (5) and anti-inflammatory (6) effects. Recent studies demonstrate that SP can prevent heart injury by inhibiting mitochondrial damage, oxidative stress, and endoplasmic reticulum stress (7–9). However, the effects of SP in DCM induced by T2D still need further investigation.

In the present study, we elaborated on the morphological, molecular, proteomic, and metabolomic changes in the myocardium of T2D and SP-treated mice. Research information from the study can be used to discriminate new candidate targets and pathways for SP therapy in DCM.

## Materials and methods

### Experimental animals

Homozygous 8-week-old male db/db mice (22 ± 0.5 g) on a C57BL/6 background were provided by the Animal Research Institute of Mudanjiang Medical University (MMU), and the study was approved by the MMU Medical Science Ethics Committee. All mice were maintained on a 12-h light/dark cycle and fed with a standardized chow and clean water *ad libitum*. The mice were randomly divided into three groups (*n* = 8 per group): (1) Control group: 8-week-old male C57BL/6 mice were injected with stroke-physiological saline solution buffer (SPSS); (2) T2D group: db/db mice; (3) T2D+SP group: db/db mice intraperitoneal injection of SP (10 mg/kg in SPSS), the dosing interval of animals in each group was every other day. All mice in the three groups were sacrificed in week 12, and relevant experimental indexes were detected according to the protocols.

### Serum measurements

Blood glucose was measured from the mouse tail vein blood by a blood glucose meter (ACCU-CHEK, Roche, Germany). In week 12, blood samples taken from the medial canthal vein were centrifuged and serum was used to detect relevant indicators. Serum insulin levels were determined by an ELISA kit (Tongwei, Shanghai, China). Serum levels of triacylglycerol (TG) and total cholesterol (TC) were analyzed using a standard biochemistry panel (Beyotime, Nantong, China). Lactate dehydrogenase (LDH), creatine kinase isoenzyme (CK-MB), and cardiac troponin-I (cTnI) in the blood serum were measured by using commercially available kits (Jiancheng Institute of Bioengineering, Nanjing, China). All kits were performed according to the manufacturer's instructions.

### Echocardiographic analysis

A Vivid 7 Dimension echocardiography machine was used to assess cardiac function and dimensions (MyLab Delta-vet Esaote, Italy). All the mice were anesthetized with 2 % isoflurane to perform echocardiography. Left ventricular ejection fraction (EF), left ventricular fractional shortening (FS), left ventricular internal dimension systole (LVIDs), and left ventricular internal dimension diastole (LVIDd) were measured.

### Histology analysis

After anesthesia, the heart was quickly excised and washed with pre-cooled PBS buffer. The cardiac tissue was fixed in 10 % buffered paraformaldehyde, embedded in paraffin, sliced at 4 millimeters, and subjected to histological staining for morphological observation. Hematoxylin and eosin staining (HE), Masson trichrome staining, and Sirius red staining were conducted according to the protocol, and the stained sections were observed using a computational color image analysis system (Leica Microscope DM2700M, Germany).

### Transmission electron microscopy

To observe the ultrastructural changes, TEM analysis was performed as previously described (10). Briefly, heart tissues were fixed with 2.5 % glutaraldehyde overnight at 4 °C and further fixed in 1 % osmium tetroxide for 2 h. Subsequently, heart tissues were dehydrated using a graded series of ethanol, embedded in epoxy resin, and observed using an H-7650 transmission electron microscope (Hitachi, Japan).

### Protein extraction and trypsin digestion

According to the previous method (11), an appropriate amount of heart tissue samples from each group were weighed and put in a mortar pre-cooled with liquid nitrogen. The liquid nitrogen was added to fully grind to powder, and the samples were added with four times the volume of powder lysis buffer (8 M urea, 1 % protease inhibitor), ultrasonically lysed, and centrifuged at 12,000 *g* for 10 min at 4 °C to remove cell debris, and transferred the supernatant to a new centrifuge tube. The protein concentration was measured using a BCA kit.

Equal amounts of each sample protein were taken for enzymatic hydrolysis, and the volumes were adjusted to the same volume with lysis buffer. The final concentration of 20 % TCA was slowly added, mixed by vortex, and precipitated at 4 °C for 2 h. The samples were centrifuged at 4,500 *g* for 5 min at 4 °C, and then, the supernatant was discarded. The precipitates were washed 2–3 times with pre-cooled acetone. After drying the precipitate, added TEAB (200 mM) was added and further subjected to ultrasonication. Samples were treated with trypsin at a ratio of 1:50 for enzymolysis overnight. The next day, dithiothreitol (DTT) of 5 mM was added to the sample and maintained at 56 °C conditions for 30 min. Finally, iodoacetamide (IAA, 11 mM) was added and incubated at room temperature for 15 min in the dark.

### LC–MS/MS analysis

The peptides were dissolved in phase A of the liquid chromatography mobile phase and separated using EASY-nLC 1,200 ultra-high performance liquid phase system (Thermo Fisher Scientific). Mobile phase A was an aqueous solution containing 0.1 % formic acid and 2 % acetonitrile; mobile phase B was an aqueous solution containing 0.1 % formic acid and 90 % acetonitrile. Liquid phase gradient settings are as follows: 0–68 min, 6–23% B; 68–82 min, 23–32% B; 82–86 min, 32–80% B; 86–90 min, 80% B, and the flow rate was maintained at 500 nL/min. Peptides were separated by an ultra-high performance liquid phase system and injected into an NSI ion source for ionization before entering the Orbitrap Exploris™ 480 mass spectrometer for analysis.

### Database search and quantification of selected proteins

Mass spectral data were retrieved using Proteome Discoverer (v2.4.1.15). Searching database is Mus_musculus_10090_SP_20201214.fasta (17,063 sequences). The digestion method was set to Trypsin (Full); the number of missed cleavage sites was set to 2; the minimum length of the peptide segment was set to six amino acid residues; the maximum number of peptide modifications was set to three; primary precursor ion mass error tolerance was set to 10 ppm, and mass error tolerance for secondary fragment ions was 0.02 Da. Carbamidomethyl (C) was set as fixed modification; the oxidation (M), acetyl (N-terminus), Met-loss (M), Met-loss+acetyl (M), and deamidated (N, Q) were set as variable modification. Proteins, peptides, and FDRs identified by PSM were all set to 1 %.

### Bioinformatic analysis

Fold change of DEPs > 1.30 or < 0.77 and a *P* < 0.05 were set as the significant thresholds. A volcano plot and a hierarchical clustering analysis were carried out using the Cluster 3.0 software. KEGG enrichment analysis, enrichment of gene ontology (GO) biological process, cellular component, and molecular function terms were analyzed using David's Functional Annotation Chart tool (Version 6.8) (12, 13).

### Western blot analysis

The mouse heart tissues were homogenized in 0.5 ml of RIPA (1:1000 PMSF, Solarbio Science, Beijing) buffer using small tubes and vibrated every 10 min for 5 s at 4°C, and the above process was repeated four times. Solubilized proteins were centrifugated at 13,500 rpm for 25 min, and the supernatant was then collected. The protein concentration of each sample was quantified using the BCA Protein Assay kit (Beyotime, Shanghai). Protein lysates of each group were separated by electrophoresis with SDS-PAGE and electro-transferred onto a PVDF membrane (Millipore). Non-specific proteins on membranes were blocked with 5% non-fat dried milk for 2 h at room temperature, and the membranes were incubated overnight with the following primary antibodies (at a 1:1000 dilution, 4 °C): PRKG1, Ppm1k, Pltp, Hacd2, (ABclonal Technology, Wuhan, China); Pir (Abcam Technology, USA); Thbs1, (Cell Signaling Technology, Danvers, MA); Tmem70, β-tubulin (Santa Cruz Biotechnology). Then, the membranes were incubated with anti-mouse/anti-rabbit IgG antibody (ABclonal Technology, Wuhan, China) at a 1:10000 dilution for 1 h at room temperature. The specific complex was determined by an enhanced chemiluminescent (ECL) kit (Meilun, Dalian, China) and a multiplex fluorescent imaging system (ProteinSimple, CA).

### Statistical analyses

Statistical significance was determined using Fisher's exact test with Benjamini–Hochberg's corrected *P* < 0.05. Hierarchical clustering analysis was conducted for the DEPs, based on the significant enrichments, using the “heatmap.2” function from the “gplots” R package. Statistical analysis was performed by the two-tailed Student's *t-*test or the one-way ANOVA, followed by the Bonferroni multiple comparison test using GraphPad Prism 9.0, and a *P* < 0.05 was considered statistically significant.

## Results

### SP improves cardiac functions in db/db mice

**Figure 1:**
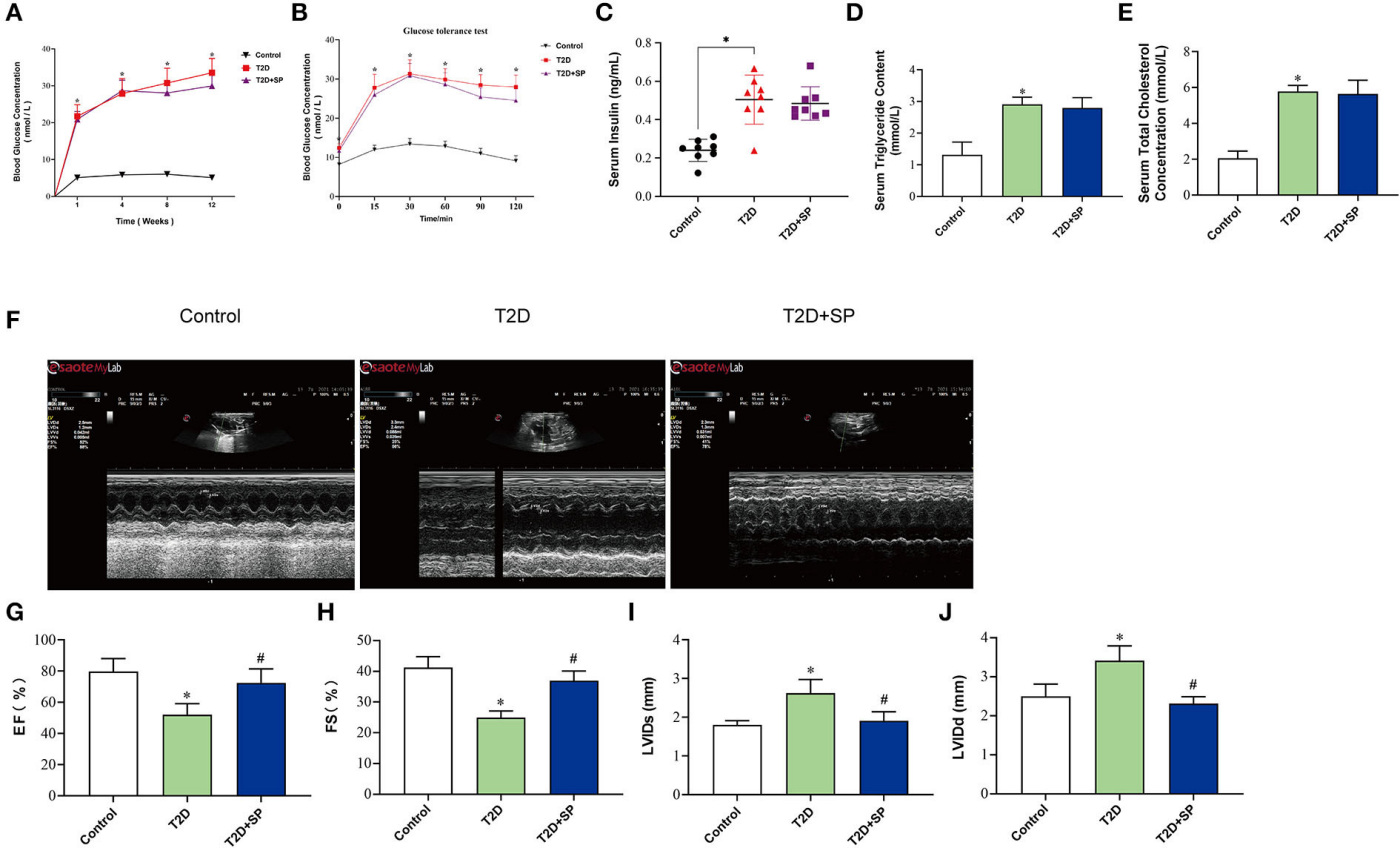
SP improves cardiac functions in the T2D mouse model. Db/db mouse was selected as the research object of the T2D model, and the related indexes were determined at week 12. **(A)** Blood glucose concentration, **(B)** insulin resistance test, **(C)** serum insulin concentration, **(D)** triacylglycerol, **(E)** total cholesterol, **(F)** echocardiography, **(G)** left ventricular ejection fraction, **(H)** left ventricular fractional shortening, **(I)** left ventricular internal dimension systole, and **(J)** left ventricular internal dimension diastole *P < 0.05 *vs*. Control group; ^#^*P* < 0.05 *vs*. T2D group (*n* = 8).

To better observe the effects of SP, we selected 8-week-old wild-type mice and db/db mice as our research objects and intraperitoneally injected with SP every other day for 12 weeks. Glucose levels, glucose intolerance, insulin levels, triglyceride (TG), and total cholesterol (TC) levels were examined on week 12, which recapitulated the hallmark features of type II diabetes. The results showed that compared with the Control group, the blood glucose levels and glucose intolerance in the T2D group at each time point were higher. While the insulin level was observed as significantly increased, TG and TC measurements were obviously increased in the T2D group. Interestingly, compared with the T2D group, there were no differences in the above indexes found in the T2D+SP group (Figures 1A–E).

To determine whether SP improved heart function in db/db mice, echocardiography was performed. Compared with the Control group, we observed that both ejection fraction (EF) and left ventricular fractional shortening (FS) were decreased in the T2D group, and the left ventricular internal dimension systole (LVIDs) and left ventricular internal dimension diastole (LVIDd) were increased. Such changes were found inverted in the T2D+SP group compared to the T2D group (Figures 1F–J).

### SP reduces myocardium damage and collagen deposition in T2D animal model

**Figure 2:**
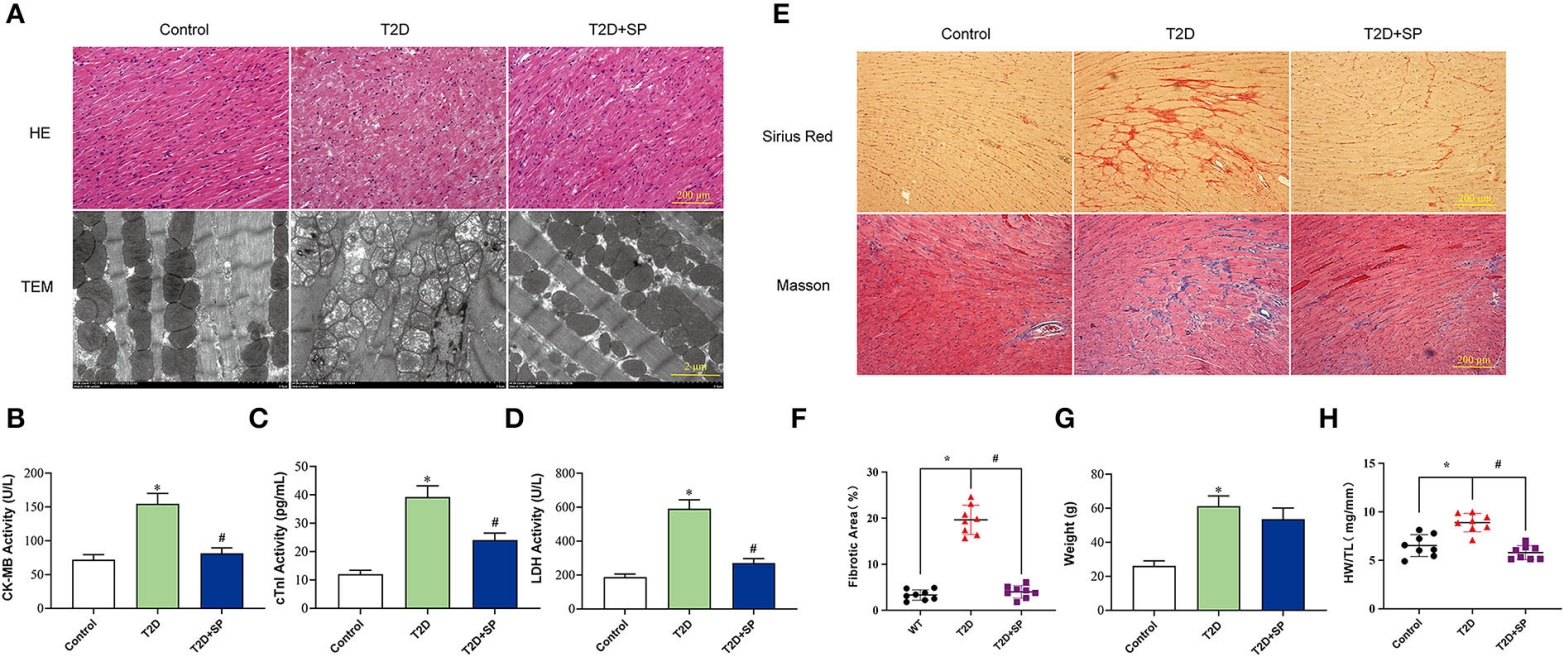
SP ameliorates diabetic cardiomyopathy by protecting myocardial tissue and reducing collagen depositions. **(A)** Representative images of H&E staining and transmission electron microscopy, **(B)** serum CK-MB concentration, **(C)** serum cTnI concentration, **(D)** serum lactate dehydrogenase concentration, **(E)** representative Sirius red staining and Masson's trichrome of cross sections of heart tissues, **(F)** fibrotic area statistics of cardiac tissue, **(G)** body weight, and **(H)** the ratio of heart weight to tibia length. *P < 0.05 *vs*. Control group; ^#^*P* < 0.05 *vs*. T2D group (*n* = 8).

To verify that diabetic cardiomyopathy can cause serious cardiac injury, we detected morphological changes in the collected mice myocardium tissues. HE staining showed that the cardiac myocytes were presenting a disordered and hypertrophic morphology in db/db mice, TEM further showed myocardial myofilament lysis and mitochondrial edema, and SP could mitigate these changes (Figure 2A).

The detections of the serum myocardial injury markers showed that the contents of serum CK-MB, cTnI, and LDH in the T2D group were significantly higher than those in the Control group. Compared with the T2D group, the serum contents of the above enzymes in the T2D+SP group were found significantly decreased. These indicated that SP has protective effects on myocardial injury caused by high glucose (Figures 2B–D).

Masson and Sirius Red staining results revealed larger amounts of collagen deposition in the T2D group compared to the Control group. The body weight (BW) and the ratio of heart weight to tibia length (HW/TL) of db/db mice on week 12 were higher than the wild-type mice. Compared with the T2D group, SP reversed these changes except for the BW (Figures 2E–H).

### Identification and cluster analysis of differentially expressed proteins in heart tissues

**Figure 3:**
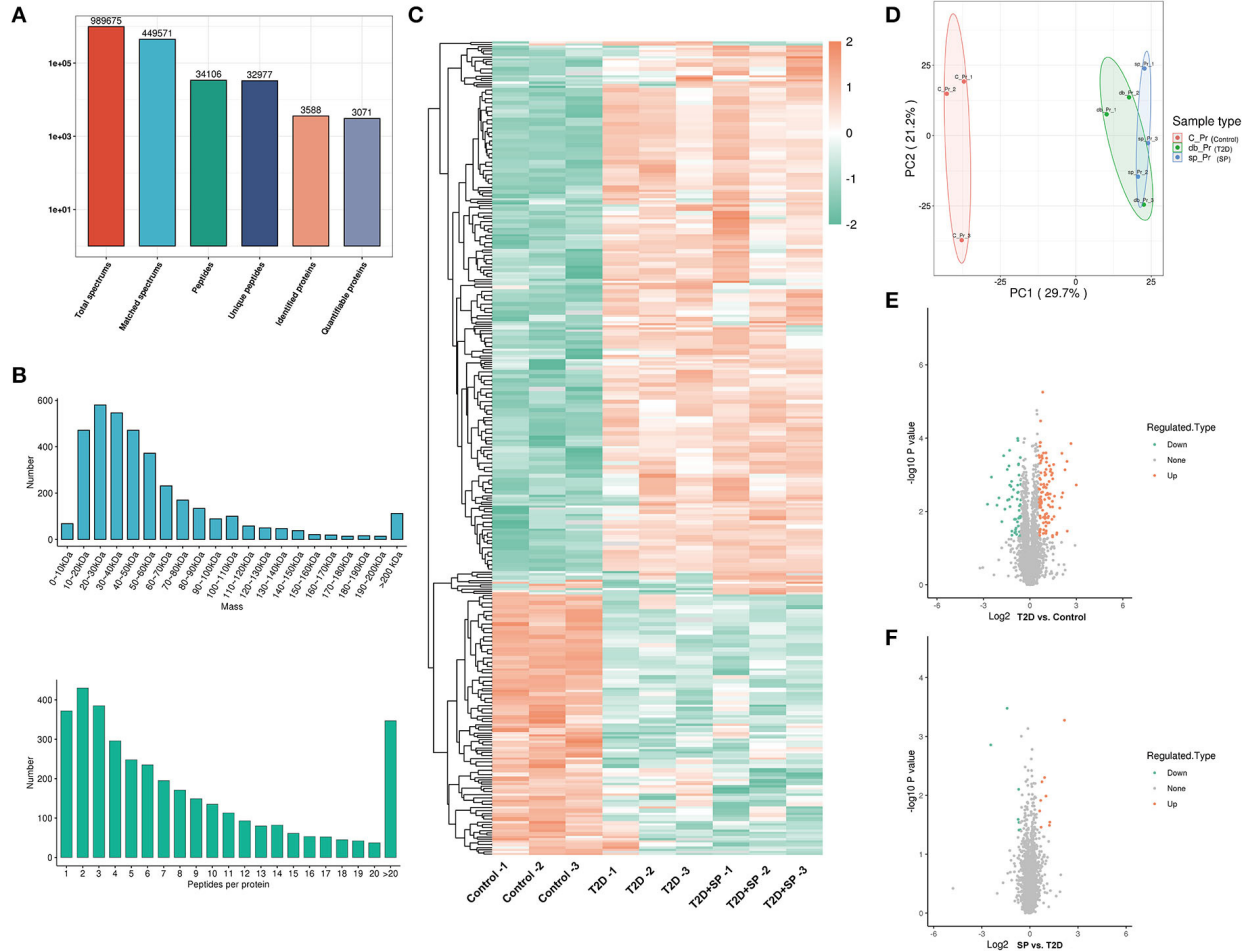
Identification of differentially expressed proteins (DEPs) using mass spectrometry. **(A)** Bar graph summarizing the detected peptides and proteins, **(B)** distribution of detected peptides and proteins, **(C)** heatmap of DEPs in the heart tissues of the mice, **(D)** PCA of the proteomics data, the points represent different samples, **(E)** volcano plot showing the quantitative protein expression in cardiac tissues from C57BL/6 mice and db/db mice, DEPs with fold change > 1.5 are marked in color, and **(F)** volcano plot showing the quantitative protein expression of cardiac tissues between the T2D and T2D+SP groups, DEPs with fold change > 1.5 are marked in color.

The heart tissues in the assigned groups were subjected to mass spectrometry analysis, and 989,675 secondary spectrograms were obtained totally. The number of available effective spectrograms was 449,571, and the utilization rate of the spectrograms was 45.4 %. A total of 34,106 peptides were identified by spectrogram analysis, among which 32,977 were specific peptide segments. A total of 3,588 proteins were identified, among which 3,071 were quantifiable and most of the protein molecular weights were 20–60 kDa (Figures 3A,B). By using a detailed heat-mapping method, DEPs were analyzed using a hierarchical cluster algorithm, and different colors represented the expression levels of DEPs in the heat map. Compared with the Control group, a great number of protein expressions in the T2D group were altered (Figures 3C,D). A total of 169 DEPs were identified in the T2D/Control group, including 115 upregulated and 54 downregulated proteins. Furthermore, 16 DEPs were appraised in the T2D+SP/T2D group and the volcano plot summarized these results (Figures 3E,F, Table 1).

**Table 1:** Differentially expressed proteins between the T2D+SP group and T2D group.

**Protein ID**	**Gene name**	**Fold change of T2D**	***p*_Value**	**Regulated type**
P49962	Srp9	0.6029	0.025	Down
P35441	Thbs1	0.1844	0.001	Down
Q921N7	Tmem70	0.6245	0.028	Down
Q8BGN2	D3Ertd751e	2.0051	0.01	Up
Q9D3B1	Hacd2	0.6555	0.038	Down
Q9D711	Pir	1.6818	0.005	Up
P01869	Ighg1	2.3321	0.032	Up
P55065	Pltp	2.3713	0.028	Up
Q8C9H6	Strip2	1.6238	0.034	Up
P08074	Cbr2	1.5969	0.012	Up
Q6DFX2	Antxr2	0.3741	0.0003	Down
P0C605	Prkg1	0.6162	0.0079	Down
O08582	Gtpbp1	1.8851	0.004	Up
Q6NVF9	Cpsf6	4.4406	0.0005	Up
Q8BXN7	Ppm1k	0.6243	0.039	Down
P54729	Nub1	1.5266	0.018	Up

### Functional enrichment analysis of the DEPs

**Figure 4:**
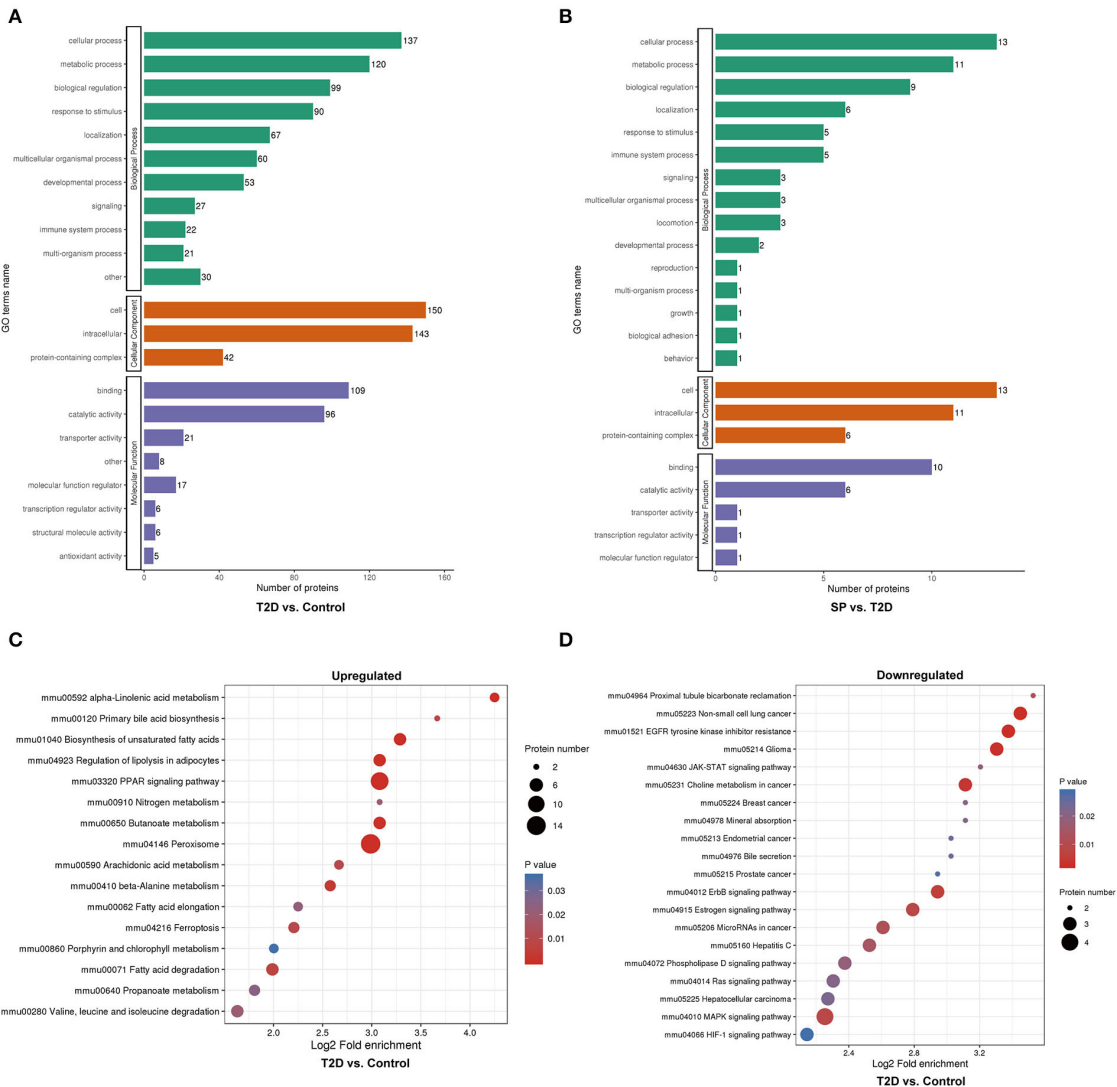
Gene ontology (GO) analysis and Kyoto Encyclopedia of Genes and Genomes (KEGG) pathway enrichment analysis of the differentially expressed proteins (DEPs). **(A)** GO enrichment analysis of the DEPs between Control and T2D groups, **(B)** GO enrichment analysis of the DEPs between T2D and T2D+SP groups, **(C)** bubble diagrams representing the top 16 KEGG pathways where upregulated DEPs enriched, and **(D)** bubble diagrams representing the top 20 KEGG pathways where downregulated DEPs enriched.

Functional enrichment analysis was performed on the DEPs based upon the Gene Ontology (GO) and Kyoto Encyclopedia of Genes and Genomes (KEGG) reference databases. Markedly, the DEPs in the T2D/Control group and the T2D+SP/T2D group were significantly enriched in several relevant GO terms, respectively, including metabolic process, biological regulation, developmental process, immune system process, and transporter activity (Figures 4A,B). The DEPs between the T2D group and the Control group were enriched in multiple KEGG pathways. Specifically, several metabolism-related pathways were enriched, such as fatty-acid degradation (mmu00071), butanoate metabolism (mmu00650), peroxisome (mmu04146), ferroptosis (mmu04216), PPAR signaling pathway (mmu03320), and biosynthesis of unsaturated fatty acids (mmu01040). Furthermore, the MAPK signaling pathway was enriched and a large proportion of the DEPs related to this pathway were downregulated (Figures 4C,D).

### Hierarchical cluster analysis of protein expression of heart tissue from WT mice and db/db mice

**Figure 5:**
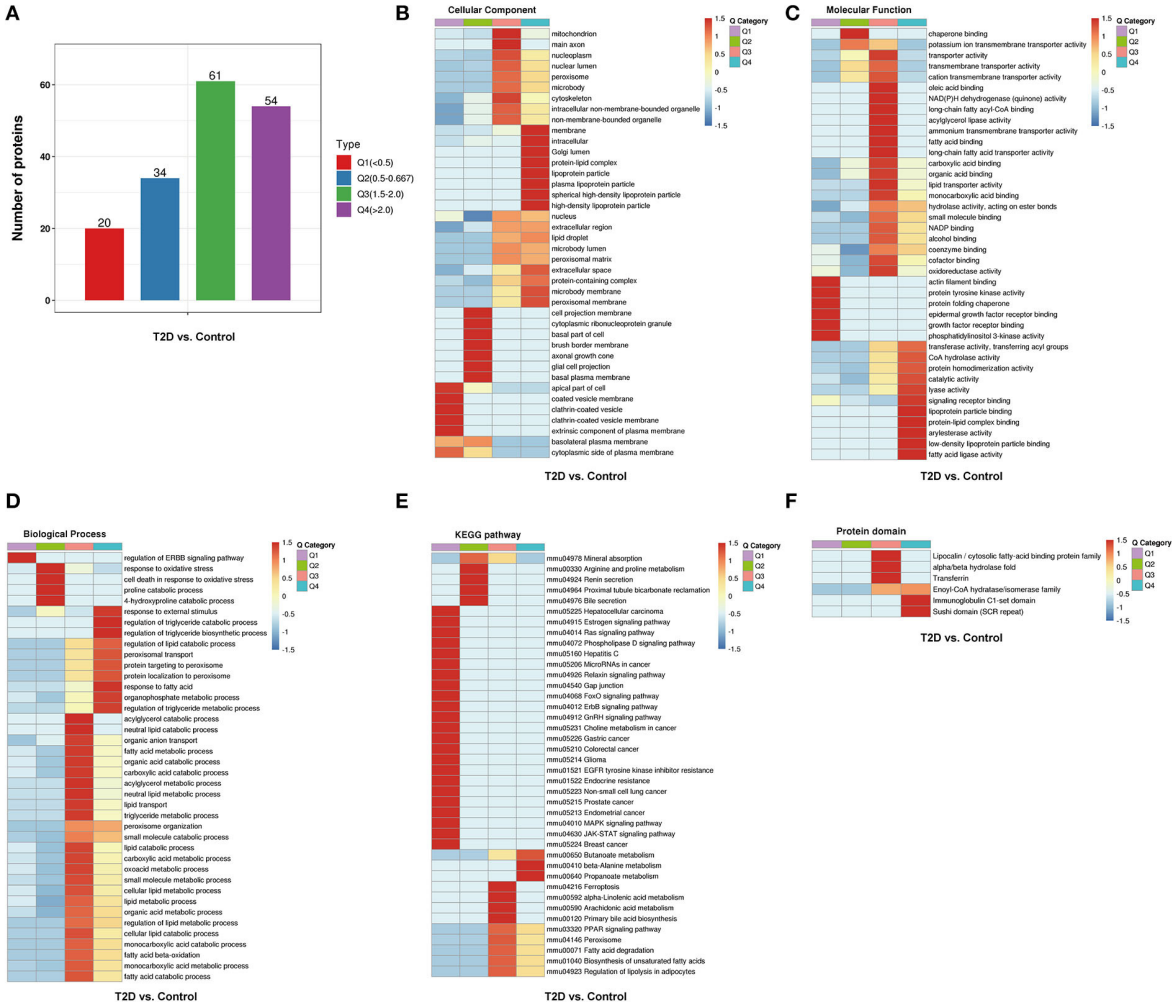
Hierarchical cluster analysis of DEPs from the WT and db/db mouse heart tissue. **(A)** According to the degree of fold change (FC), the DEPs were categorized into four groups from Q1 to Q4 (Q means the ratio of db/db mouse group to C57BL/6 mouse group, Q1 < 0.5, Q2 = 0.5–0.667, Q3 = 1.5–2, and Q4 > 2), **(B)** cellular component, **(C)** molecular function, **(D)** biological process, **(E)** the Kyoto Encyclopedia of Genes and Genomes pathways, and **(F)** protein domain. The red color indicates stronger enrichment. The blue color indicates weaker enrichment.

To further study the differential protein expression in T2D mice, a hierarchical cluster analysis in db/db and WT mice groups was performed and DEPs were divided into four regions according to the degree of fold change (Figure 5A). DEPs of GO cluster analysis showed that some significantly upregulated DEPs were enriched for peroxisome function, synthesis, and metabolism of triglyceride, fatty acid, and lipid (Figures 5B–D). The common enriched pathways were shown in the functional KEGG enrichment cluster image (Figure 5E), and gap junction, ferroptosis, and JAK-STAT and MAPK signaling pathways were enriched in db/db mice compared to the WT mice. In the protein domain cluster analysis, some evidently upregulated DEPs were enriched for major lipocalin/cytosolic fatty-acid protein, enoyl-CoA hydratase/isomerase family, and Sushi domain (Figure 5F).

### The correlation analysis between proteomics and metabolomics

**Figure 6:**
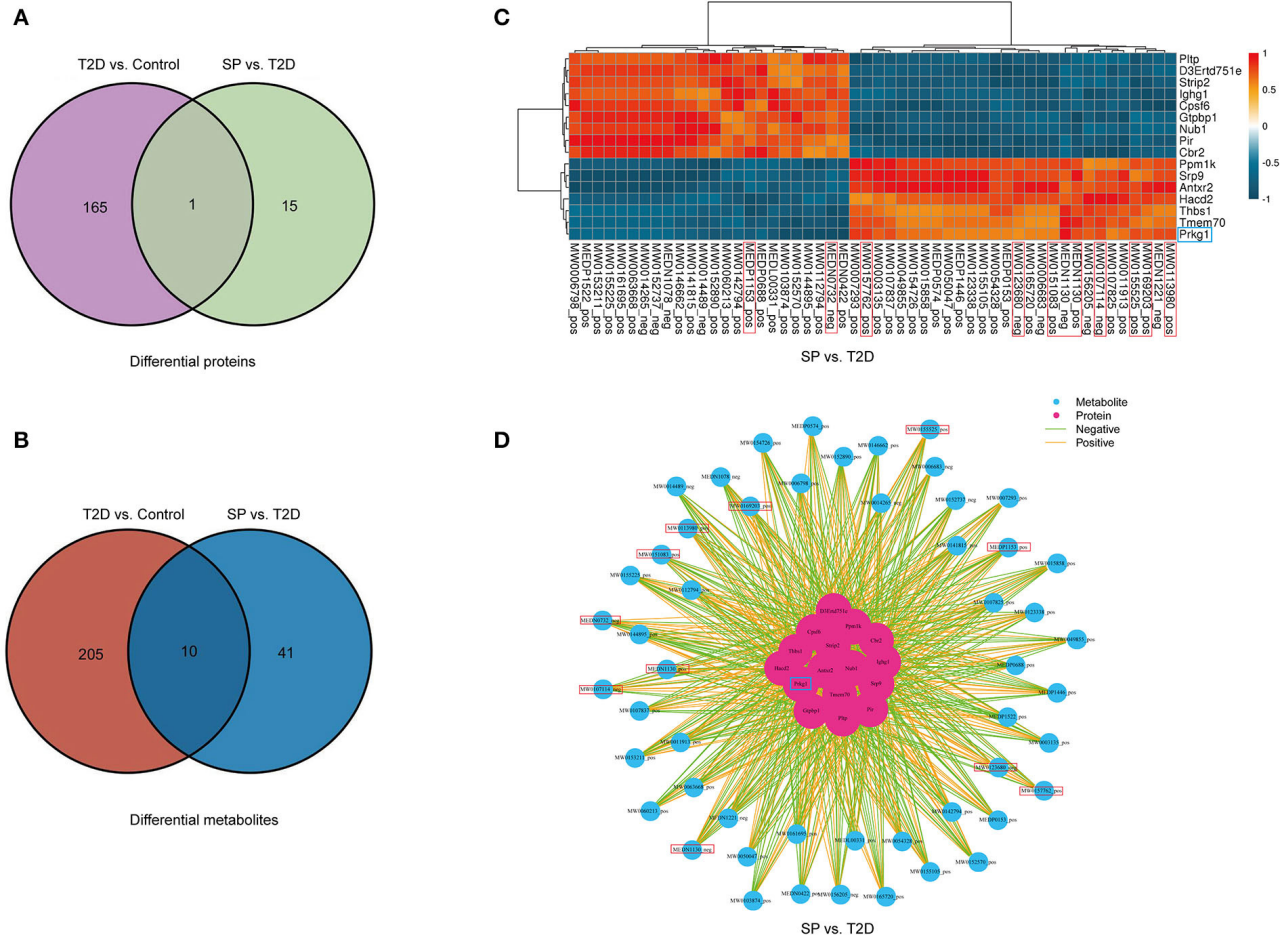
Correlation analysis of proteomics and metabolomics reveals the role of SP in DCM. **(A)** Venn diagrams displaying the one overlapped protein that is differentially expressed in both T2D/Control and T2D+SP/T2D groups. **(B)** Venn diagrams displaying the 10 overlapped metabolites that are differentially expressed in both T2D/Control and and T2D+SP/T2D groups. **(C)** The heatmap displaying the regulatory relationship of DEPs and the differentiated metabolites in db/db mice treated with SP. **(D)** Protein–metabolite correlation network analysis in db/db mice treated with SP.

**Figure 7:**
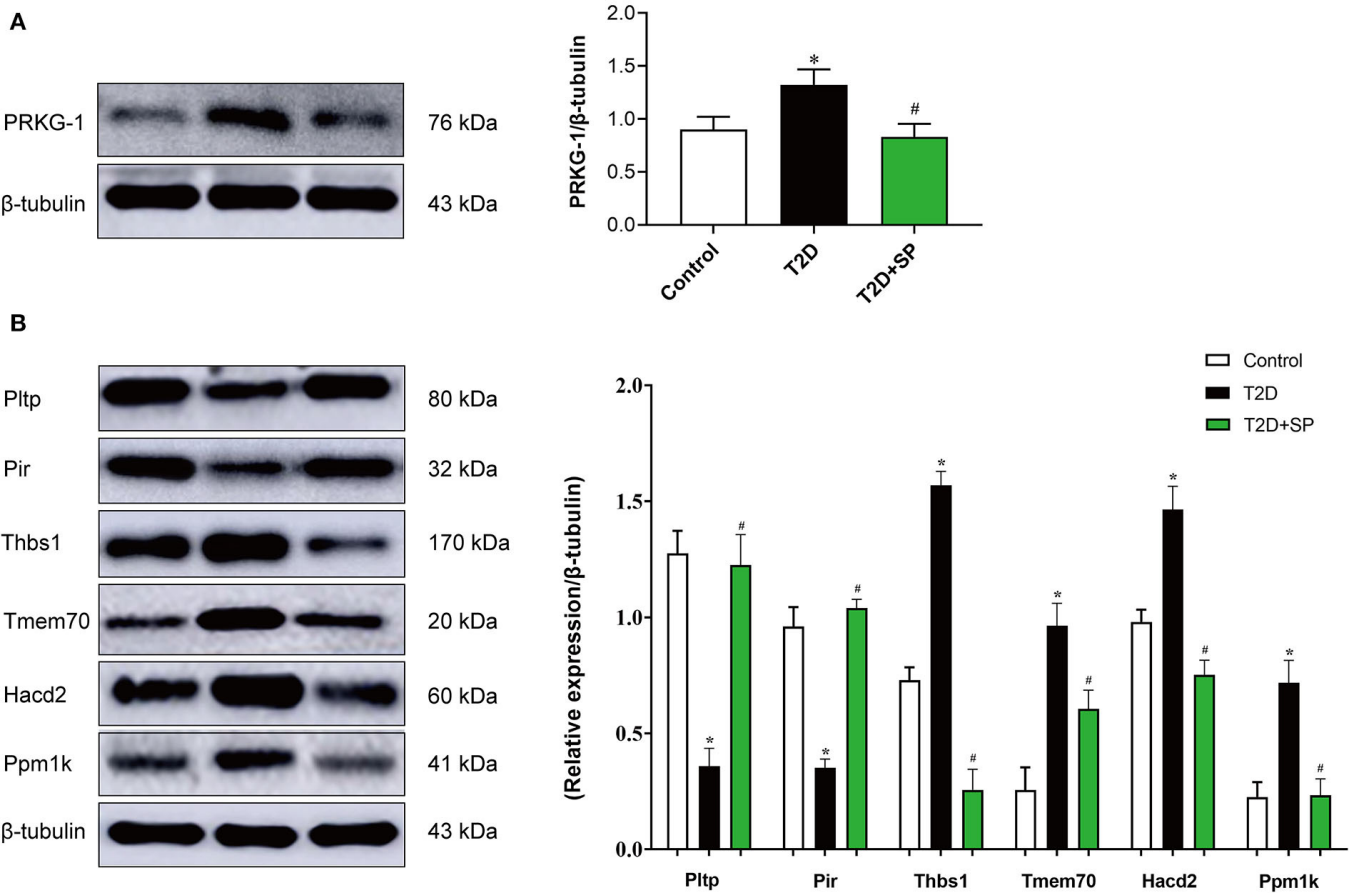
Western blot analysis of the DEPs between the db/db and SP-treated mice. **(A)** The protein expression level of PRKG1 was determined by the Western blot. **(B)** The protein expression levels of Pltp, Pir, Thbs1, Tmem70, Hacd2, and Ppm1k were detected by Western blot. *P < 0.05 *vs*. Control group; ^#^*P* < 0.05 *vs*. T2D group (*n* = 8).

Through data analysis of DEPs in each group, there was only 1 (PRKG1) DEP was found presenting in both the T2D+SP/T2D group and the T2D/Control group (Figure 6). Metabolomic analysis results showed that SP could regulate 51 differential metabolites in db/db mice, among which 10 differentiated metabolites were found to overlap between the T2D+SP/T2D group and the T2D/Control group (Figure 6B, Table 2). Further correlation analysis demonstrated that PRKG1 could regulate the 10 differential metabolomics (Figures 6C,D). Furthermore, Western blot results proved that the expression of PRKG1 was upregulated in the T2D group and downregulated in the T2D+SP group (Figure 7A). Subsequently, we examined the expression of six DEPs in the T2D+SP/T2D group. The expression of Pltp and Pir was downregulated, while Thbs1, Tmem70, Hacd2, and Ppm1k expressions were upregulated in the T2D group compared to the Control group. The opposite trends were found in the T2D+SP group compared with the T2D group (Figure 7B).

**Table 2:** Differential metabolites between the T2D+SP group and T2D group.

**Index**	**Compounds**	**Formula**	***p*_Value**	**Molecular weight**	**Ion mode**	**Type**
MW0014489	4-Hydroxyacid	C4H8O3	0.022	104.042	[M-H]^−^	Up
MEDN1221	D-Mannose 6-phosphate	C6H13O9P	0.03	259.021	[M-H]^−^	Down
MW0152737	Lobelanine	C22H25NO2	0.044	354.188	[M-H]^−^	Up
MEDN1078	cis-EODA	C18H34O3	0.031	297.233	[M-H]^−^	Up
MEDN0732	12-HOME	C18H34O3	0.0009	297.233	[M-H]^−^	Up
MW0014265	3-Oxostearic acid	C18H34O3	0.04	297.242	[M-H]^−^	Up
MW0123680	Dihydroretrofractamide B	C22H31NO3	0.012	392.193	[M-H]^−^	Down
MW0156205	4-t-Amylphenol	C11H16O	0.022	163.112	[M-H]^−^	Down
MW0107114	Glycerylphosphorylethanolamine	C5H14NO6P	0.036	429.107	[M-H]^−^	Down
MW0006683	Cyflufenamid	C20H17F5N2O2	0.012	411.116	[M-H]^−^	Down
MEDN1130	NADH	C21H29N7O14P2	0.046	647.116	[M-H]^−^	Down
MW0060213	PE-NMe2(14:1(9Z)/24:1(15Z))	C45H86NO8P	0.005	877.530	[M-H]^+^	Up
MEDP1153	20-HETE	C20H32O3	0.031	286.224	[M-H]^+^	Up
MW0113980	beta-D-Fructose 2-phosphate	C6H13O9P	0.043	278.063	[M-H]^+^	Down
MEDN0422	2'-Deoxyadenosine	C10H13N5O3	0.002	274.091	[M-H]^+^	Up
MW0161695	[(E)-3-(4-hydroxy-3-methoxyphenyl) prop-2-enyl] (E)-8-methylnon-6-enoate	C20H28O4	0.041	315.190	[M-H]^+^	Up
MW0154726	Oleate	C18H34O2	0.012	247.242	[M-H]^+^	Down
MW0049855	DG (16:1(9Z)/18:0/0:0)	C37H70O5	0.012	577.517	[M-H]^+^	Down
MEDP0574	Oleamide	C18H35NO	0.044	563.549	[M-H]^+^	Down
MEDP1446	Octadecadienamide	C18H33NO	0.022	280.263	[M-H]^+^	Down
MEDL00331	3'-Aenylic Acid	C10H14N5O7P	0.032	695.128	[M-H]^+^	Up
MW0050047	DG (18:0/24:1(15Z)/0:0)	C45H86O5	0.016	791.620	[M-H]^+^	Down
MW0165720	(1R,2E,7S,8R)-9-hydroxy-1,8-dimethyl-12-propan-2-yltricyclo[9.3.0.03,7]tetradeca-2,11-diene-4-carbaldehyde	C20H30O2	0.029	387.192	[M-H]^+^	Down
MW0144895	Allopurinol riboside	C10H12N4O5	0.044	269.088	[M-H]^+^	Up
MW0006798	Dicyclohexyl phthalate	C20H26O4	0.047	295.164	[M-H]^+^	Up
MW0153211	Lys Val Tyr Lys	C26H44N6O6	0.032	537.331	[M-H]^+^	Up
MW0015858	Arachidonoyl amide	C20H33NO	0.022	304.261	[M-H]^+^	Down
MEDP0688	Benzophenone	C13H10O	0.049	183.080	[M-H]^+^	Up
MW0063668	Sterebin B	C20H32O5	0.031	397.193	[M-H]^+^	Up
MW0142794	3alpha,7alpha-Dihydroxy-12-oxo-5beta-cholanate	C24H38O5	0.0003	835.528	[M-H]^+^	Up
MEDP0153	3-Methylxanthine	C6H6N4O2	0.047	167.055	[M-H]^+^	Down
MW0155225	Phe Ile Gln Lys	C26H42N6O6	0.044	535.315	[M-H]^+^	Up
MW0155105	Phe Asn Glu	C18H24N4O7	0.022	409.174	[M-H]^+^	Down
MW0141815	1-O-Palmitoyl-2-O-acetyl-sn-glycero-3-phosphorylcholine	C26H52NO8P	0.045	538.348	[M-H]^+^	Up
MW0151083	His Ser Leu Ser Glu	C23H37N7O10	0.011	572.271	[M-H]^+^	Down
MW0103874	(2S)-2-Hydroxybutanedioic acid	C4H6O5	0.033	172.976	[M-H]^+^	Up
MW0003135	2-Hydroxybenzamide	C7H7NO2	0.023	297.083	[M-H]^+^	Down
MW0054328	Leukotriene E4	C23H37NO5S	0.047	481.269	[M-H]^+^	Down
MW0107825	Leukotriene D4 methyl ester	C26H42N2O6S	0.008	528.307	[M-H]^+^	Down
MW0112794	3-Deoxy-D-glycero-D-galacto-2-non-ulosonic acid	C9H16O9	0.04	291.071	[M-H]^+^	Up
MW0146662	beta-D-Mannosyl-1,4-N-acetyl-D-glucosamine	C14H25NO11	0.022	430.156	[M-H]^+^	Up
MW0155525	Phosphonoformyl-CMP	C10H15N3O12P2	0.015	473.044	[M-H]^+^	Down
MW0152570	Leu Val Thr Leu Ala	C24H45N5O7	0.039	516.343	[M-H]^+^	Up
MW0107837	Leupeptin	C20H38N6O4	0.002	495.283	[M-H]^+^	Down
MW0157762	Thr Phe Ile	C19H29N3O5	0.044	402.197	[M-H]^+^	Down
MW0152890	Lys Asn Ser	C13H25N5O6	0.017	392.147	[M-H]^+^	Up
MW0169203	galangin	C15H10O5	0.023	339.050	[M-H]^+^	Down
MW0123338	Cinoxacin	C12H10N2O5	0.012	263.069	[M-H]^+^	Down
MW0011913	1,2-Dipalmitoleoyl-sn-glycero-3-phosphocholine	C40H76NO8P	0.026	774.479	[M-H]^+^	Down
MW0007293	Haloxyfop	C15H11ClF3NO4	0.013	761.013	[M-H]^+^	Down
MEDP1522	Carnitine C13:0	C20H39NO4	0.006	404.299	[M-H]^+^	Up

## Discussion

Diabetes mellitus(DM)is a metabolic disease, often classified into type I and type II. While the former accounts for about 10% of all cases of diabetes and is usually induced by congenital pancreatic islet dysfunction, the more prevalent form (90%), type II, is mostly caused by insulin resistance (14, 15). According to clinical research statistics, type II diabetes has a much higher incidence of cardiovascular disease than type I diabetes (16). Therefore, it is critical to study the pathogenesis and treatment strategies of type II diabetic cardiomyopathy.

In the present study, we selected db/db mouse, a model of type II diabetes as the research object, whose genetic background is C57BL/6 mouse diabetes gene knocked out and even insulin intervention fails to control hyperglycemia. Therefore, db/db mice are widely used in the study of endocrine defects, neurological diseases, and cardiac diseases induced by abnormal glucose and lipid metabolism. At 1, 4, 8, and 12 weeks, db/db mouse showed persistent hyperglycemia, and there was no downward trend following SP injection. At week 12, insulin activity, and serum TC and TG levels increased significantly in the T2D group. The result was consistent with the trends in the insulin resistance test, which indicated that the type II diabetes mouse model was successfully established. However, SP had no effect on the above indicators.

After 12 weeks of modeling, echocardiography displayed decreased EF and FS and increased LVIDs and LVIDd in the T2D group, indicating heart systolic dysfunction. Further myocardial injury marker enzyme test confirmed increased levels of LDH, CK-MB, and cTnI in the blood serum, and SP could attenuate the above indicators of heart damage. In the T2D group and the T2D+SP group, db/db mice showed a significant increase in body weight; however, treating with SP could decrease the HW/TL ratio. We suspected the change was due to heart remodeling and also increasing the myocardial extracellular matrix. This speculation is supported by cardiac morphology. In the T2D group, heart weight increased, Masson and Sirius red staining showed large amounts of collagen deposition in the interstitial areas, TEM results presented obvious myofilament dissolution and mitochondrial edema, and HE results showed db/db mouse heart eventuated degeneration and cardiomyocytes necrosis. In our findings, SP could inhibit the above changes effectively. To further evaluate the effects of SP on T2D mice, we performed relevant omics studies to discover the underlying molecular regulatory mechanisms.

To explore the features of T2D mice with SP treatment, the characteristics and differences of each group were comprehensively analyzed using the 4D label-free techniques. We identified 169 DEPs between db/db and C57BL/6 mice, and only 16 DEPs have been identified in the T2D group treated with SP. This shows that the heart is a comparably stable organ with a relatively steady protein expression. To further clarify the protective mechanism of SP on the heart, the DEPs of the T2D/Control group were subjected to GO analysis. It was found that most of the proteins identified in the BP category were closely related to the cellular process, the metabolic process, and the biological regulation, while the lipid and fatty-acid metabolic processes were involved the most. We also subjected the DEPs to the KEGG pathways. The results showed that these annotated differentiated proteins were mainly related to the PPAR signaling pathway, peroxisome, gap junction, ferroptosis pathway, and MAPK signaling pathway. From the above data analysis, the differentiated proteins were found to be associated with lipid metabolism and amino acid metabolism; therefore, we performed the following metabolomic analysis.

Metabolomics, a systematic research method, is a newly developed discipline after genomics and proteomics. It quantitatively analyzes all metabolites in the organism and finds the relative relationship between metabolites and physiological and pathological changes. The heart tissue metabolites of each group that were differentially expressed among C57BL/6 mice, db/db mice, and db/db mice treated with SP were examined using untargeted metabolomics techniques. A total of 205 and 51 differentially metabolites were identified in the T2D/Control group and T2D+SP/T2D group, respectively. Among these, 10 overlapping differentiated metabolites were identified between the two groups. These metabolites were characterized, belonging to flavonoids, amino acids, fructose phosphate, glycerolipid, and amides, and were closely related to diabetes-induced cardiovascular diseases (17–21). More interestingly, the expression trends of these metabolites could be reversed by SP treatment.

In our study, proteomics revealed that PRKG1 was the only differentially expressed protein in both the T2D/Control group and the T2D+SP/T2D group. The expression of PRKG1 was found significantly upregulated in the heart tissue of db/db mice, and SP treatments could substantially suppress its expression. The above-mentioned changes have been further verified by the Western blot analysis. Furthermore, through proteomic and metabolomic association analysis, we found that PRKG1 was closely related to the expressions of 10 overlapping metabolites between the T2D/Control and the T2D+SP/T2D group. Evidence from existing research data shows that PRKG1 (cGMP-dependent protein kinase-1) is a serine/threonine-specific protein kinase, involving in the conduction of nitric oxide/cGMP signaling pathway, and has the functions of relaxing smooth muscle, inhibiting platelet aggregation and regulating cell growth (22, 23). Recent research data prove that PRKG1 can regulate the cardiovascular system, endocrine system, and nervous system (24). The above studies reveal that PRKG1 plays a crucial role in the occurrence and progression of DCM.

We used proteomic-based data-independent acquisition to further analyze the DEPs in the T2D+SP/T2D group. We noticed that six DEPs (Pltp, Pir, Thbs1, Tmem70, Hacd2, and Ppm1k) closely related to cardiovascular disease were either significantly downregulated or upregulated. Pltp (phospholipid transfer protein), a monomeric protein that belongs to a family of lipid transfer/lipopolysaccharide-binding proteins, involves in lipoprotein metabolism, cardiovascular diseases, and atherosclerosis, as well as obesity and insulin resistance (25–27). Pir (Pirin), an iron-dependent redox regulator of NF-κB, can regulate the autophagy-dependent ferroptosis process in eukaryotic cells (28, 29). Thbs1 (Thrombospondin-1) has been certified that its overexpression can lead to lethal cardiac atrophy (30). Transmembrane protein 70 (Tmem70), a mitochondrial protein, located in the inner membrane of mitochondria, is closely related to ATP synthesis of encephalo-cardiomyopathy (31). 3-hydroxyacyl-CoA dehydratase 2 (Hacd2), a member of the HACD family, has been investigated in obesity and metabolic diseases (32). Moreover, recent studies have confirmed that protein phosphatase 1K (Ppm1k) can elevate the risk of cardiovascular disease (33). Hereafter, the expression of the above 6 DEPs was verified by the Western blot, and the results were found consistent with the omics results. Although our study screened out many differentiated proteins that were closely related to the pathogenesis of DCM, their specific roles need to be further explored one by one in future experiments.

Taken as a whole, our proteomic and metabolomic data indicate that SP may play a vital role in the type II DCM treatment process. Our results verify potential research candidates, such as PRKG1, Pltp, Pir, Thbs1, Tmem70, Hacd2, and Ppm1k, which could be further evaluated as therapeutic targets. In addition, our findings can be a potential source of information for the treatment strategy of DCM in the future.

## Data availability statement

The datasets presented in this study can be found in online repositories. The names of the repository/repositories and accession number(s) can be found at: Proteomics identifications database (PRIDE database) with accession PXD036364.

## Ethics statement

The animal study was reviewed and approved by Animal Research Institute of Mudanjiang Medical University.

## Author contributions

HY conceived and supervised the study, analyzed data, and wrote the manuscript. HY and JS designed experiments. HY, YLin, JS, JX, XX, YHao, YHan, and SZ performed experiments. YLiu provided new tools and reagents. FL edited language for the manuscript. All authors reviewed the results and approved the final version of the manuscript.

## Funding

This research was supported by the Basic Scientific Research Business Research Project of Heilongjiang (No.2021-KYYWF-0466) and the Doctoral Research Start-up Fund Project of Mudanjiang Medical University (No.2021-MYBSKY-040).

## Conflict of interest

The authors declare that the research was conducted in the absence of any commercial or financial relationships that could be construed as a potential conflict of interest.

## Publisher's note

All claims expressed in this article are solely those of the authors and do not necessarily represent those of their affiliated organizations, or those of the publisher, the editors and the reviewers. Any product that may be evaluated in this article, or claim that may be made by its manufacturer, is not guaranteed or endorsed by the publisher.

## Abbreviations

DCM: diabetic cardiomyopathy
SP: spermine
DEPs: differentially expressed proteins
PAs: polyamines
TC: total cholesterol
TG: triglyceride
HE: hematoxylin and eosin
LVIDs: left ventricular internal dimension systole
LVIDd: left ventricular internal dimension diastole
EF: left ventricular ejection fraction
FS: left ventricular fractional shortening.

## References

[1] Rubler S Dlugash J Yuceoglu YZ Kumral T Branwood AW Grishman A. New type of cardiomyopathy associated with diabetic glomerulosclerosis. Am J Cardiol. (1972) 30:595–602. 10.1016/0002-9149(72)90595-4 4263660

[2] Parim B Sathibabu Uddandrao V Saravanan G. Diabetic cardiomyopathy: molecular mechanisms, detrimental effects of conventional treatment, and beneficial effects of natural therapy. Heart Fail Rev. (2019) 24:279–99. 10.1007/s10741-018-9749-1 30349977

[3] Cai Z Yuan S Luan X Deng L Li J Feng J. Pyroptosis-related inflammasome pathway: a new therapeutic target for diabetic cardiomyopathy. Front Pharmacol. (2022) 13:842313. 10.3389/fphar.2022.842313 35355717 PMC8959892

[4] Wang J Li S Wang J Wu F Chen Y Zhang H. Spermidine alleviates cardiac aging by improving mitochondrial biogenesis and function. Aging. (2020) 12:650. 10.18632/aging.102647 31907336 PMC6977682

[5] Zou T Rao JN Guo X Liu L Zhang HM Strauch ED. Nf-Kappab-Mediated Iap Expression Induces Resistance of Intestinal Epithelial Cells to Apoptosis after Polyamine Depletion. Am J Physiol Cell Physiol. (2004) 286:C1009–18. 10.1152/ajpcell.00480.2003 15075199

[6] Jeong J-W Cha H-J Han MH Hwang SJ Lee D-S Yoo JS. Spermidine protects against oxidative stress in inflammation models using macrophages and zebrafish. Biomol Ther. (2018) 26:146. 10.4062/biomolther.2016.272 28365977 PMC5839493

[7] Chai N Zhang H Li L Yu X Liu Y Lin Y. Spermidine prevents heart injury in neonatal rats exposed to intrauterine hypoxia by inhibiting oxidative stress and mitochondrial fragmentation. Oxid Med Cell Longev. (2019) 2019:5406468. 10.1155/2019/5406468 31217839 PMC6537013

[8] Wang Y Chen J Li S Zhang X Guo Z Hu J. Exogenous spermine attenuates rat diabetic cardiomyopathy via suppressing ros-p53 mediated downregulation of calcium-sensitive receptor. Redox Biol. (2020) 32:101514. 10.1016/j.redox.2020.101514 32234613 PMC7113441

[9] Wang Y Wang Y Li F Zhang X Li H Yang G. Spermine protects cardiomyocytes from high glucose-induced energy disturbance by targeting the casr-Gp78-ubiquitin proteasome system. Cardiovasc Drugs Ther. (2021) 35:73–85. 10.1007/s10557-020-07064-z 32918657

[10] Yuan H Xu J Zhu Y Li L Wang Q Yu Y. Activation of calcium-sensing receptor-mediated autophagy in high glucose-induced cardiac fibrosis *in vitro*. Mol Med Rep. (2020) 22:2021–31. 10.3892/mmr.2020.11277 32705187 PMC7411369

[11] Buckley JP Quirós-Alcalá L Teitelbaum SL Calafat AM Wolff MS Engel SM. Associations of prenatal environmental phenol and phthalate biomarkers with respiratory and allergic diseases among children aged 6 and 7 years. Environ Int. (2018) 115:79–88. 10.1016/j.envint.2018.03.016 29550712 PMC5970077

[12] Sun Y Zhang L Lu B Wen J Wang M Zhang S. Hydrogen sulphide reduced the accumulation of lipid droplets in cardiac tissues of Db/Db Mice via Hrd1 S-sulfhydration. J Cell Mol Med. (2021) 25:9154–67. 10.1111/jcmm.16781 34562065 PMC8500968

[13] Zhang Y Zhang S Li B Luo Y Gong Y Jin X. Gut microbiota dysbiosis promotes age-related atrial fibrillation by lipopolysaccharide and glucose-induced activation of Nlrp3-inflammasome. Cardiovasc Res. (2022) 118:785–97. 10.1093/cvr/cvab114 33757127

[14] Association AD. Diagnosis and classification of diabetes mellitus. Diabetes Care. (2010) 33(Suppl. 1):S62–9. 10.2337/dc10-S062 20042775 PMC2797383

[15] Severson DL. Diabetic cardiomyopathy: recent evidence from mouse models of type 1 and type 2 diabetes. Can J Physiol Pharmacol. (2004) 82:813–23. 10.1139/y04-065 15573141

[16] Rawshani A Rawshani A Franzén S Eliasson B Svensson A-M Miftaraj M. Mortality and cardiovascular disease in type 1 and type 2 diabetes. N Engl J Med. (2017) 376:1407–18. 10.1056/NEJMoa1608664 28402770

[17] Jankauskas SS Kansakar U Varzideh F Wilson S Mone P Lombardi A. Heart failure in diabetes. Metabolism. (2021) 125:154910. 10.1016/j.metabol.2021.154910 34627874 PMC8941799

[18] Aloud AA Veeramani C Govindasamy C Alsaif MA El Newehy AS Al-Numair KS. Galangin, a dietary flavonoid, improves antioxidant status and reduces hyperglycemia-mediated oxidative stress in streptozotocin-induced diabetic rats. Redox Report. (2017) 22:290–300. 10.1080/13510002.2016.1273437 28030991 PMC6837547

[19] Myers RW Baginsky WF Gattermeir DJ Geissler WM Harris G. Enzymatic preparation of high-specific-activity ?-D-[6, 6′-3h] fructose-2, 6-bisphosphate: application to a sensitive assay for fructose-2, 6-bisphosphatase. Anal Biochem. (2010) 406:97–104. 10.1016/j.ab.2010.06.017 20541516

[20] Rhooms SK Murari A Goparaju NSV Vilanueva M Owusu-Ansah E. Insights from drosophila on mitochondrial complex I. Cell Mol Life Sci. (2020) 77:607–18. 10.1007/s00018-019-03293-0 31485716 PMC7289077

[21] Isse FA El-Sherbeni AA El-Kadi AOS. The multifaceted role of cytochrome P450-derived arachidonic acid metabolites in diabetes and diabetic cardiomyopathy. Drug Metab Rev. (2022) 54:141–60. 10.1080/03602532.2022.2051045 35306928

[22] Münzel T Feil R Mülsch A Lohmann SM Hofmann F Walter U. Physiology and pathophysiology of vascular signaling controlled by cyclic guanosine 3′, 5′-cyclic monophosphate–dependent protein kinase. Circulation. (2003) 108:2172–83. 10.1161/01.CIR.0000094403.78467.C3 14597579

[23] Zeng Y Pan Y Liu H Kang K Wu Y Hui G. Mir-20a regulates the Prkg1 gene by targeting its coding region in pulmonary arterial smooth muscle cells. FEBS Lett. (2014) 588:4677–85. 10.1016/j.febslet.2014.10.040 25447536

[24] Seftel A. Downregulation of Cgmp-dependent protein kinase-1 activity in the corpus cavernosum smooth muscle of diabetic rabbits. J Urol. (2005) 173:928. 10.1097/01.ju.0000152127.87796.9f 15711334

[25] Tzotzas T Desrumaux C Lagrost L. Plasma phospholipid transfer protein (Pltp): Review of an emerging cardiometabolic risk factor. Obes Rev. (2009) 10:403–11. 10.1111/j.1467-789X.2009.00586.x 19413703

[26] Cavusoglu E Marmur JD Chhabra S Hojjati MR Yanamadala S Chopra V. Elevated baseline plasma phospholipid protein (Pltp) levels are an independent predictor of long-term all-cause mortality in patients with diabetes mellitus and known or suspected coronary artery disease. Atherosclerosis. (2015) 239:503–8. 10.1016/j.atherosclerosis.2015.02.017 25710294 PMC4361262

[27] Chen X Sun A Zou Y Ge J Kamran H Jiang X-C. High Pltp activity is associated with depressed left ventricular systolic function. Atherosclerosis. (2013) 228:438–42. 10.1016/j.atherosclerosis.2013.02.032 23545183

[28] Liu F Rehmani I Esaki S Fu R Chen L de Serrano V. Pirin is an iron-dependent redox regulator of Nf-?b. Proc Nat Acad Sci. (2013) 110:9722–7. 10.1073/pnas.1221743110 23716661 PMC3683729

[29] Hu N Bai L Dai E Han L Kang R Li H. Pirin is a nuclear redox-sensitive modulator of autophagy-dependent ferroptosis. Biochem Biophys Res Commun. (2021) 536:100–6. 10.1016/j.bbrc.2020.12.066 33373853

[30] Vanhoutte D Schips TG Vo A Grimes KM Baldwin TA Brody MJ. Thbs1 induces lethal cardiac atrophy through perk-Atf4 regulated autophagy. Nat Commun. (2021) 12:1–16. 10.1038/s41467-021-24215-4 34168130 PMC8225674

[31] Karbanová VH Vrbacká AC Hejzlarová K Nusková H Stránecký V Potocká A. Compensatory upregulation of respiratory chain complexes III and IV in isolated deficiency of Atp synthase due to tmem70 mutation. Biochim Biophys Acta. (2012) 1817:1037–43. 10.1016/j.bbabio.2012.03.004 22433607

[32] Wei L Weng S Lu X Zhu S Yang Q Chen YQ. 3-hydroxyacyl-coa dehydratase 2 deficiency confers resistance to diet-induced obesity and glucose intolerance. Biochem Biophys Res Commun. (2022) 605:134–40. 10.1016/j.bbrc.2022.03.057 35325655

[33] Hu W Liu Z Yu W Wen S Wang X Qi X. Effects of Ppm1k Rs1440581 and Rs7678928 on serum branched-chain amino acid levels and risk of cardiovascular disease. Ann Med. (2021) 53:1317–27. 10.1080/07853890.2021.1965204 34382495 PMC8366658

